# Multifunctional Nanoplatform for NIR-II Imaging-Guided Synergistic Oncotherapy

**DOI:** 10.3390/ijms242316949

**Published:** 2023-11-29

**Authors:** Qingluo Wang, Guoyu Xia, Jianmin Li, Longlong Yuan, Shujie Yu, Dingyang Li, Nan Yang, Zhongxiong Fan, Jinyao Li

**Affiliations:** Xinjiang Key Laboratory of Biological Resources and Genetic Engineering, College of Life Science and Technology & Institute of Materia Medica, Xinjiang University, Urumqi 830017, China; 107552203673@stu.xju.edu.cn (Q.W.); xgy1823@stu.xju.edu.cn (G.X.); 107552201047@stu.xju.edu.cn (J.L.); 20191106232@stu.xju.edu.cn (L.Y.); 107552303714@stu.xju.edu.cn (S.Y.); 107552301091@stu.xju.edu.cn (D.L.); 107552303710@stu.xju.edu.cn (N.Y.)

**Keywords:** nanomaterials, second near-infrared windows, tumor imaging, synergistic oncotherapy, integration of diagnosis and treatment

## Abstract

Tumors are a major public health issue of concern to humans, seriously threatening the safety of people’s lives and property. With the increasing demand for early and accurate diagnosis and efficient treatment of tumors, noninvasive optical imaging (including fluorescence imaging and photoacoustic imaging) and tumor synergistic therapies (phototherapy synergistic with chemotherapy, phototherapy synergistic with immunotherapy, etc.) have received increasing attention. In particular, light in the near-infrared second region (NIR-II) has triggered great research interest due to its penetration depth, minimal tissue autofluorescence, and reduced tissue absorption and scattering. Nanomaterials with many advantages, such as high brightness, great photostability, tunable photophysical properties, and excellent biosafety offer unlimited possibilities and are being investigated for NIR-II tumor imaging-guided synergistic oncotherapy. In recent years, many researchers have tried various approaches to investigate nanomaterials, including gold nanomaterials, two-dimensional materials, metal sulfide oxides, polymers, carbon nanomaterials, NIR-II dyes, and other nanomaterials for tumor diagnostic and therapeutic integrated nanoplatform construction. In this paper, the application of multifunctional nanomaterials in tumor NIR-II imaging and collaborative therapy in the past three years is briefly reviewed, and the current research status is summarized and prospected, with a view to contributing to future tumor therapy.

## 1. Introduction

At present, antitumor therapy remains a worldwide challenge. The mortality and morbidity rates of tumors are very high all over the world, and they continue to rise yearly [1,2,3]. The clinical treatments for tumors mainly include chemotherapy, surgery, and radiotherapy, but the treatment effect is very unsatisfactory, characterized by strong side effects, low specificity, a high recurrence rate, drug resistance, etc. [4,5]. This cruel reality pushes people to find new and more effective treatment methods. In recent years, phototherapy (photodynamic therapy and photothermal therapy), immunotherapy (IMT) and gas therapy (GT) have been rapidly developed [6,7,8]. However, single treatment systems often fail to achieve the expected results for several reasons, such as the fact that the internal environment of solid tumors is so complex that it is a low-pH hypoxic zone, which greatly reduces the sensitivity of the tumor cells to a single anticancer drug, resulting in a much lower anticancer effect. Multimodal synergistic therapy provides higher tumor-treatment efficiency, which can even protect normal tissues from unintentional damage and prevent tumor recurrence while eliminating tumor cells. However, for some deeply buried tumors (e.g., osteosarcoma, etc.), it is often difficult to accurately reach the tumor site with anticancer drugs alone, and there is an urgent need for noninvasive imaging techniques to diagnose the disease and identify the focal area [9,10,11]. Therefore, the development of a combined treatment strategy that combines diagnosis and therapy has become a hot topic in the field of tumor treatment today [12,13,14].

Tumor photoacoustic imaging (PAI) and fluorescence imaging (FLI) have received much attention in recent years. PAI is an emerging nondestructive biomedical imaging technology that combines the properties of optical imaging and ultrasound imaging with the advantages of high spatial resolution and high contrast [15]. Once the endogenous luminophores or exogenous contrast agent has entered the tumor site, the PAI illuminates the tumor site with a nonionizing, safe laser pulse that triggers thermal expansion of the target and generates a photoacoustic (PA) wave. The PA wave can be captured by a clinical ultrasound detector (transducer) and the image processed using an ultrasound machine, facilitating the potential translation of PAI into the clinic [16]. Conventional PA contrast agents consist of endogenous chromophores (e.g., collagen, hemoglobin, melanin, cytochromes, lipids, and water) and exogenous contrast agents, which absorb light mainly in the visible (wavelengths of 400–750 nm) or the first near-infrared window (NIR-I, wavelengths of 750–1000 nm). Exogenous PA contrast agents have strong near-infrared (NIR) absorption and their absorption spectra can be distinguished from the background [17]. FLI, as a powerful in vivo imaging tool, not only provides accurate localization of drugs and direct visualization of tumors, but also spatiotemporal control and noninvasive treatment, and is widely used in clinical decision making and treatment in endoscopy or surgery [18]. Compared with other imaging modalities, such as magnetic resonance imaging (MRI), computed tomography (CT), and positron emission tomography, FLI is characterized by high spatiotemporal resolution, low cost, noninvasiveness, and good biosafety [19]. Although the advantages of PAI and FLI are outstanding, conventional contrast agents are mainly used for imaging by visible light or NIR-I laser. Biological tissues (e.g., fat, skin, and blood) absorb and scatter incident light to varying degrees, thereby reducing the depth and contrast of PAI and FLI [20]. NIR-II light (wavelength 1000–1700 nm), with its longer wavelength and weaker interaction with biological tissues can be a good solution to this problem. In addition, recent studies have shown that compared with NIR-I light, NIR-II light has lower photon dispersion, less tissue absorption, higher sensitivity, relatively higher maximum permissible skin exposure (MPE), more optimal tissue penetration depth (up to 3.5 mm), and better spatial resolution during laser irradiation [21,22]. Therefore NIR-II-responsive tumor imaging has more important applications in biological and medical fields.

**Figure 1:**
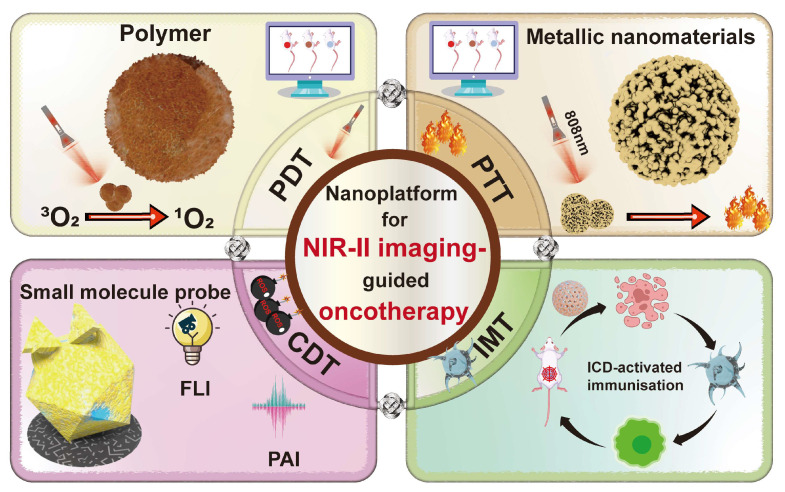
Scheme illustrating in Multifunctional Nanoplatform for NIR-II imaging-guided synergistic oncotherapy.

Despite the enormous potential of tumor imaging-guided tumor therapy, the implementation of this strategy urgently requires the development of new materials with high biosafety, high sensitivity, high resolution, and real-time and bright imaging. In recent years, noninvasive tumor therapies based on multifunctional nanomaterials with low damage to normal cells and high efficacy have been rapidly developing in the diagnosis, imaging, and treatment of tumors. Due to the optical properties of nanomaterials, such as high molar extinction coefficient (MEC), low quantum yield, good light stability and NIR absorption, the composite nanoplatform assembled by nanomaterials and contrast agents can improve the resolution and perfectly combine tumor imaging and treatment, so people have been increasingly exploring this option. In addition, nanomaterials have the advantages of longer blood circulation time, higher tumor absorption efficiency, and a wider selection of species [23]. Therefore, the development of novel nanomaterials for NIR-II PAI and FLI is a hot topic. So far, various inorganic materials (e.g., precious metals [24], two-dimensional nanomaterials [25], carbon-based nanomaterials [26], rare earth-doped nanoparticles (NPs) [27] and metal oxides [28], etc.) and organic materials (e.g., small molecular probes [29], conjugated polymers [30], semiconducting polymers [31], etc.) have been widely developed as NIR-II-responsive nanomaterials. A wide variety of nanomaterials can be used in many popular tumor therapies (Figure 1). In photodynamic therapy (PDT), polymeric nanomaterials containing photosensitizers (PSs) can damage tumor cells by producing cytotoxic reactive oxygen species (ROS), such as hydroxyl radicals (·OH) and singlet oxygen (^1^O_2_), under light irradiation. In photothermal therapy (PTT), photothermal agents (PTAs), such as some metal nanomaterials, can damage tumor cells by generating heat. In chemodynamic therapy (CDT), chemotherapeutic drugs can kill cancer cells by catalyzing endogenous hydrogen peroxide (H_2_O_2_) to produce highly oxidized ·OH through Fenton/Fenton-like reaction, but chemotherapeutic drugs often bring strong systemic toxicity. Therefore, the controlled release of chemotherapeutic drugs can be achieved by wrapping light-sensitive small molecule fluorescent probes and can be monitored in real time through in vivo imaging. In IMT, some nanomaterials induce immunogenic death (ICD) of cancer cells, typically by releasing calretin (CRT), high-mobility group protein 1(HMGB1), and adenosine triphosphate (ATP) outside the cell, thereby recruiting immune cells for IMT. It is expected that the development of a multifunctional composite nanoplatform has realized the combination tumor therapy under the guidance of tumor imaging. The composite nanoplatform may contain both inorganic and organic nanomaterials. It can be applied to PDT, PTT, etc., and has a very broad application prospect. In this paper, we review the latest research progress of nanomaterials for NIR-II imaging and combined tumor therapy in the past three years, intending to provide a reference for the subsequent development and exploitation of NIR-II-responsive diagnostic and therapeutic integrated nanoplatforms (Table 1).

## 2. Photothermal Therapy Synergized with Photodynamic Therapy

PTT and PDT have emerged as promising strategies for the treatment of tumors compared to conventional treatments. Phototherapy, a treatment that uses light to selectively ablate tumor cells with minimal invasiveness, is widely classified as PTT and PDT. Under light irradiation, PTAs or PSs damage tumor cells by generating heat and cytotoxic ROS, respectively [58]. According to the type of ROS generated, PDTs can be divided into two types, including type I PDTs and type II PDTs, which kill tumor cells by generating ·OH and ^1^O_2_, respectively. However, most PSs have poor water solubility and low oxygen production [59,60], so the efficiency of relying on PDT alone to treat tumors is very low. The heat generated by PTT can accelerate blood circulation, thus increasing the oxygen content in tumors to help oxygen-dependent type II PDT [61,62], while the ROS generated in PDT can destroy heat shock protein activity, making tumor cells more sensitive to heat [63]. The synergistic strategy of PDT and PTT has the potential to reduce trauma, minimize toxic side effects, have broad applicability and be used in palliative care [64,65]. Recent studies have shown that PDT and PTT can even disrupt the intracellular redox balance, leading to mitochondrial and even nuclear DNA damage and leakage into the cytoplasm, which activates the cGAS-STING signaling pathway and elicits an antitumor immune response with less toxic side effects [66]. However, the implementation of this combined therapeutic strategy is very challenging, as complex components are required to obtain multifunctional performance, and even two or more lasers of different wavelengths are needed. Therefore, it is crucial to investigate the construction of nanoplatforms for combined PDT and PTT therapy for their further application in biomedical fields.

### 2.1. Polymers

**Figure 2:**
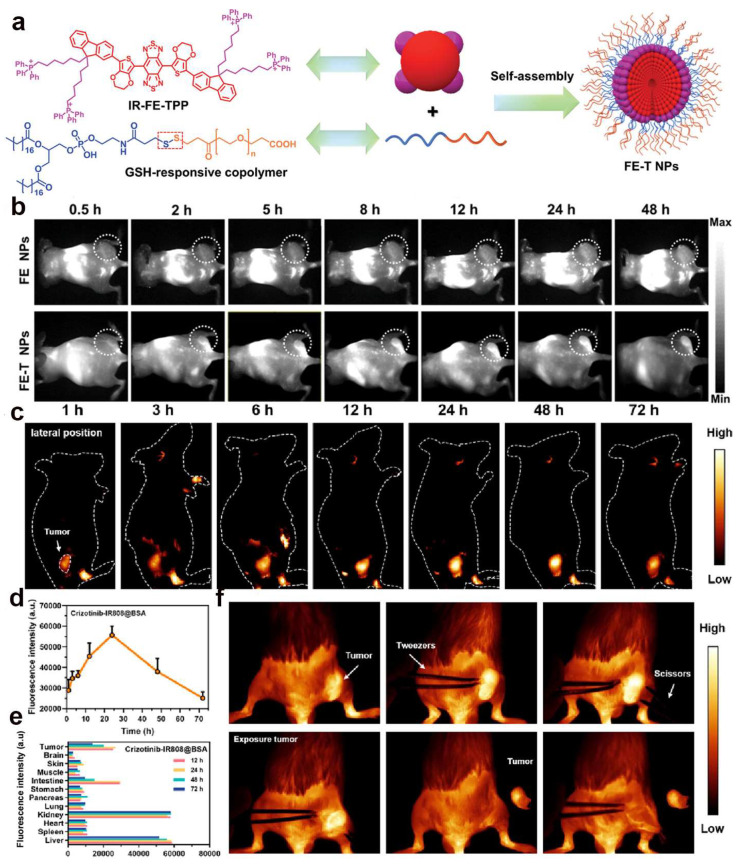
(**a**) Schematic illustration of multifunctional phototherapeutic nanoplatform FE-T NPs for single 808 nm laser-triggered NIR-II imaging-guided mitochondrial-targeting antitumor therapy; (**b**) Real-time FLI of 4T1 tumor-bearing mice for FE NPs and FE-T NPs signals at 0.5, 2, 5, 8, 12, 24, and 48 h post-injection [32]. Copyright 2023, John Wiley and Sons; (**c**) NIR-II fluorescence images (150 ms, 1000 LP) of tumor-bearing mice at different time points after injection of Crizotinib-IR808@BSA; (**d**) Fluorescence intensity of tumor in the mice after administration of Crizotinib-IR808@BSA; (**e**) Semiquantitative analysis according to the fluorescence intensity in major organs and tumors after injection of Crizotinib-IR808@BSA; (**f**) Series images of the entire surgical process under NIR-II imaging 24 h after Crizotinib-IR808@BSA injection (150 ms, 1000 LP) [71]. Copyright 2023, American Chemical Society.

Several polymers have been shown to have good photothermal conversion efficiency and photothermal stability in NIR-II. Organic conjugated polymers are an ideal NIR-II absorbing material that can promote efficient NIR-II photothermal conversion by narrowing the NIR absorption range [67]. Although NIR-II FLI has higher spatial resolution and deeper tissue penetration, most of the fluorescent probes are in the “always-on” mode, emitting unchanged fluorescence signals in both diseased and normal tissues due to their nonspecific effects [68]. Currently, tumor localization by NIR-II FLI alone can easily lead to false positives in normal tissues. Therefore, many efforts have been devoted to the development of stimulus-responsive NIR-II nanomaterials to optimize FLI by responding to the tumor microenvironment (TME) or cellular signals, including redox, pH, enzymes, etc. Yang et al. [32] developed a mitochondria-targeted phototherapeutic agent with the ability to target mitochondria, NIR-II FLI, and synergistic PTT/PDT/IMT tumor therapeutic effects with high performance. FE-T NPs were prepared by a simple self-assembly process from glutathione (GSH)-responsive copolymer DSPE-SS-PEG-COOH and mitochondria-targeted photosensitizer IR-FE-TPP (IR-FE modified by four TPPs) (Figure 2a). The IR-FE-TPP molecule, as a mitochondria-targeted photosensitizer, was completely retained inside the NPs after being encapsulated into NPs before reaching the tumor, which is the key to achieving the amplified effect of phototherapy on tumor tissues and effective suppression of the adverse reaction in normal tissues. The disulfide in the copolymer can activate the release of IR-FE-TPP through elevated GSH expression after accumulating in the tumor through enhanced permeability and retention (EPR) effect and intracellular uptake, thus achieving precise targeting of the mitochondria. Under 808 nm laser irradiation, the molecular photosensitizer IR-FE-TPP kills tumor cells with high levels of ROS and hyperthermia as well as in situ ICD, providing strong antitumor efficacy and reducing systemic adverse effects. In addition, FE-T NPs have deep penetration imaging with NIR emission (1000–1400 nm) and show good NIR-II FLI effect in tumors, which has the potential for clinical application (Figure 2b). This diagnostic–therapeutic integrated nanoplatform provides a promising strategy for effective tumor treatment.

### 2.2. Small Molecule Probe

Small molecule probes (e.g., certain peptides and inhibitors) have unique advantages over macromolecular polymers in tumor therapy due to their convenient synthesis methods, simple chemical modifications, and strong stability [69,70]. Tao et al. [33] synthesized fluorescent probes with NIR-I emission and applied them to tissue and tumor model imaging. However, the shorter emission wavelength may limit its application in deep tissues. Hu et al. [71] covalently synthesized Crizotinib-IR808 with the clinically used drug Crizotinib and the NIR-II fluorescent dye IR808, with a unique structure that provides c-Met targeting ability, excellent ^1^O_2_ generation efficiency, and high photothermal conversion efficiency. Moreover, the optical stability and water solubility of the nanomaterials were significantly improved by the embedding of bovine serum albumin, which made it easier to specifically accumulate in colorectal cancer and allowed for a clearer differentiation of blood vessels, tumors, and peripheral boundaries by NIR-II imaging (Figure 2c–f). The NPs can be used not only to study PTT synergistic PDT therapy but also for tumor NIR-II imaging and intraoperative NIR-II image-guided tumor dissection.

Although NIR-II FLI has made significant progress in clinical disease diagnosis, tumor types and TME are diverse, and to improve the accuracy of tumor diagnosis, localization, and treatment, multiple imaging modalities to collectively detect and depict information about the tumor environment have more obvious advantages than a single modality [72]. PAI combines the advantages of optical imaging and ultrasound imaging to provide deeper tissue penetration and even tumor contouring at the microscopic level [73,74], so the integration of NIR-II FLI and PAI is more conducive to biomedical applications [75,76,77]. Yang et al. [34] developed a structured organic phototherapeutic drug (Y16-Pr) for NIR-II FLI and PAI-guided synergistic treatment of PTT and PDT, which is a “receiver-donor-Acceptor’-Donor-Acceptor” (A-DA’D-A) structured organic phototherapy drug. Y16-Pr is a thick ring structure molecule with a benzotriazole as the electron-deficient nucleus and 3-dicynoviny-lindan-1-one as the end group, which can form rigidly conjugated molecular planes to promote strong light absorption and charge transfer within the molecule. Increasing the intramolecular push–pull effect can significantly reduce the energy gap between the singlet–triplet states and improve the PDT therapeutic effect [78]. The Y16-Pr molecule has an extremely strong push–pull effect, and its aqueous solution has strong absorption in the near-infrared region (600–900 nm), with a fluorescence peak at 920 nm that extends to 1100 nm, resulting in good NIR-II PAI and FLI capabilities. In addition, the Y16-Pr-PEG NPs obtained after further modification of Y16-Pr with amphiphilic polyethylene glycol (PEG) exhibited a photothermal conversion efficiency of 82.4% and could simultaneously produce ·OH and ^1^O_2_, which is expected to be a candidate for FLI/PAI bimodal imaging-directed synergistic antitumor therapy. Yang et al. [35] synthesized a dye BTP-4F-DMO with an acceptor-donor-acceptor (A-D-A) structure and prepared it as water-soluble NPs. The obtained BTP-4F-DMO nanoparticles showed strong absorption in the range of 650–850 nm, with a fluorescence emission peak at ~900 nm up to 1100 nm. Under 808 nm laser irradiation, the nanoparticles exhibited an ultra-high photothermal conversion efficiency of 90.5 ± 5% and produced both ·OH and ^1^O_2_ with a quantum yield of 4.6% for ^1^O_2_ production. The nanomaterial is a promising multifunctional photothermal sensitizer for NIR-II fluorescence/photo-acoustic dual-mode imaging-guided synergistic PDT/PTT. In conclusion, the rapid application of organic small-molecule A-D-A analogous structural dyes provides a new strategy for diagnostic and therapeutic integrated nanoplatforms.

### 2.3. Metal Nanomaterials

**Figure 3:**
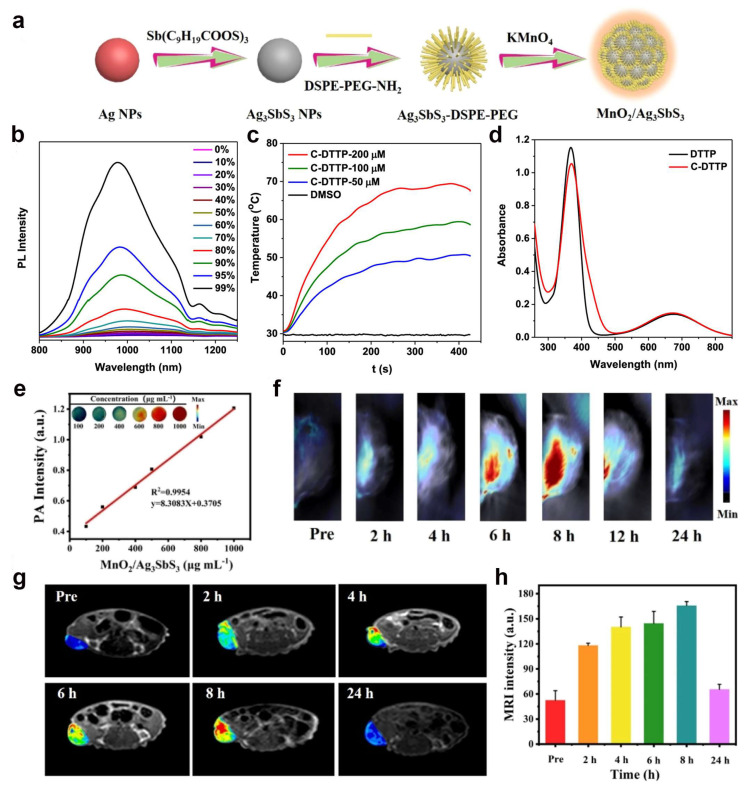
(**a**) Schematic illustration for the preparation of MnO_2_/Ag_3_SbS_3_ [37]. Copyright 2022, American Chemical Society; (**b**) Absorption spectra of DTTP and C-DTTP; (**c**) Emission spectra of C-DTTP in DMSO/toluene mixtures with different toluene fractions (*f*_T_); (**d**) Photothermal conversion behavior of C-DTTP at different concentrations under 808 nm laser irradiation (0.8 W/cm^2^) [36]. Copyright 2022, American Chemical Society; (**e**) In vitro PA images and PA signal intensity of MA solution with various concentrations; (**f**) Time-dependent PA images of 4T1 tumor-bearing mice after i.v. injection of MA at various time points; (**g**) MRI of the tumor region and (**h**) MRI intensity changes of the tumors in tumor-bearing mice pre-/post-injection of MA NPs at 2, 4, 6, 8, and 24 h [37]. Copyright 2022, American Chemical Society.

In past decades, discrete supramolecular coordination complexes (SCCs) have been widely used in catalysis, molecular sensing, and biomedicine because of their delicate and elegant structures [79,80]. A large number of metal nanomaterials with well-defined shapes and sizes have been constructed by coordination-driven self-assembly due to the availability of various metal ions and organic ligands [81,82,83,84,85]. Qin et al. [36] successfully constructed the NIR-II photoresponsive metal nanomaterial C-DTTP by assembling the four-armed ligand DTTP with aggregation-induced emission (AIE) activity and the 90° platinum receptor Pt(PEt_3_)_2_(OTf)_2_. C-DTTP has remarkable AIE properties with a maximum emission wavelength of up to 1005 nm, (Figure 3b–d), which is the longest fluorescence emission compared to the reported fluorescent SCCs. In addition, C-DTTP exhibited enhanced ROS generation and satisfactory photothermal conversion efficiency compared to the ligand DTTP. The unique features give C-DTTP unprecedented performance in NIR-II fluorescence/photothermography-guided synergistic PDT/PTT. Moreover, C-DTTP is the first example of diagnosis and treatment integration of discrete NIR-II fluorescence/photothermal imaging-guided tumor cotreatment.

Ag_3_SbS_3_ is an important narrow-band semiconductor (1.5–1.7 eV) that exhibits good light absorption in the NIR-II band (1000–1700 nm) and can be used as an ideal NIR-II photothermal converter for tumor PTT with the feasibility of PAI and high biosafety. In addition, Ag_3_SbS_3_ NPs exhibited good photosensitivity under 1064 nm light and could be used as NIR-II PDT nanoreagents. However, Ag_3_SbS_3_ is rarely used in biomedical applications. Wang et al. [37] synthesized Ag_3_SbS_3_ NPs [86,87] by a typical hydrothermal method and then modified Ag_3_SbS_3_ with PEG and loaded with MnO_2_ to form biodegradable MnO_2_/Ag_3_SbS_3_, referred to as MA (Figure 3a). In acidic TME, MA could regulate TME by catalyzing the generation of O_2_ from endogenous hydrogen peroxide (H_2_O_2_) via a Fendon-like reaction and depleting overexpressed GSH, which not only alleviated tumor hypoxia and antioxidant capacity but also improved PDT and CDT efficiency. Importantly, MnO_2_/Ag_3_SbS_3_ achieved a single laser-triggered NIR-II PTT/PDT effect under 1064 nm laser irradiation, which greatly improved the efficiency of tumor therapy and achieved tumor treatment or even tumor eradication. In addition, MnO_2_/Ag_3_SbS_3_ can be used for PAI and MRI bimodal imaging to guide treatment more accurately (Figure 3e–h).

## 3. Photothermal Therapy Synergistic Chemodynamic Therapy

PTT synergized with CDT has emerged as a promising anticancer strategy to achieve desired therapeutic effects while mitigating side effects. CDT can kill cancer cells by catalyzing the generation of highly oxidized ·OH from endogenous H_2_O_2_ via a Fenton/Fenton-like reaction, which is of great interest because of its high specificity and noninvasiveness [88,89,90,91,92]. However, the limited level of H_2_O_2_ (~100 IM) inside the tumor cannot satisfy the demand for efficient CDT, and thus, additional H_2_O_2_ supply is needed to enhance the effectiveness of CDT. In recent years, PTT triggered by NIR-II light has a broad application prospect in clinical tumor therapy due to its deep tissue heating ability and has been coupled with FLI to achieve the integration of diagnosis and treatment in the tumor region [93,94]. It is a win–win strategy for people to construct a tumor therapeutic platform by choosing some suitable NIR-II-responsive nanomaterials (e.g., polymers, metal nanomaterials, etc.), that not only can generate ROS through Fenton-like reaction but also can act as a PTA to mediate the PTT of the tumor.

### 3.1. Polymers

**Figure 4:**
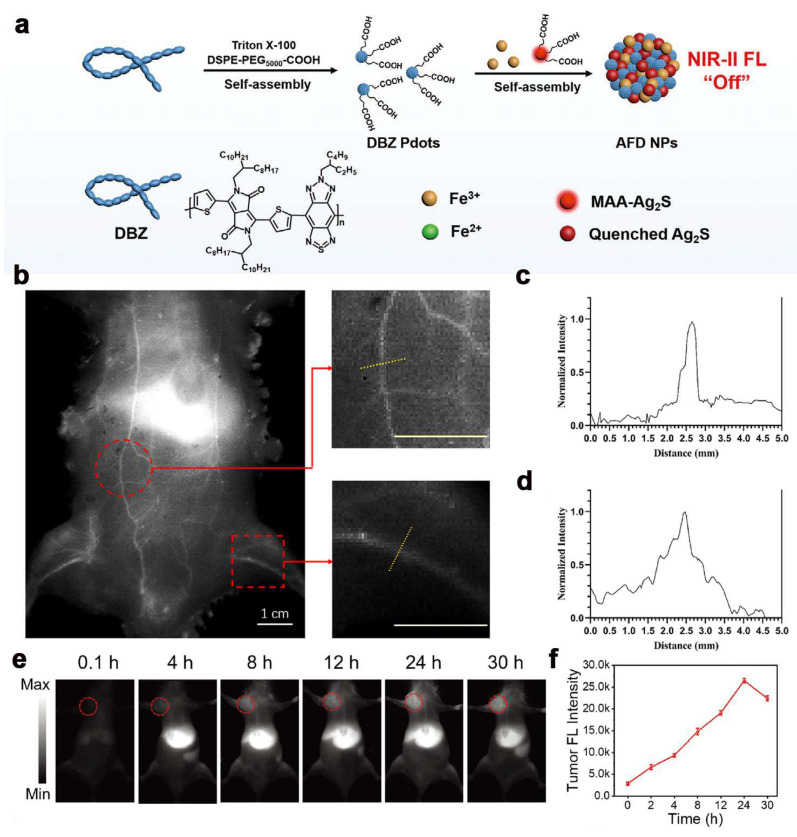
(**a**) Schematic illustration of the synthesis of TME-activated phototheranostic AFD NPs [38]. Copyright 2023, John Wiley and Sons; (**b**) Systemic angiography of the Balb/c mice body after intravenous injection with RRIALP-C4 under 1064 nm laser excitation (1064 nm LP and 1064 nm OD filters); (**c**,**d**) Fluorescence intensity analysis of abdomen and hind limb vasculatures [43]. Copyright 2023, Springer Nature; (**e**) NIR-II FLI (under 808 nm laser excitation, with 1064 nm LP filter) of mice with 143B tumors at various time intervals after tail vein injection with BTP/DOX/2DG NPs (1.0 mg mL^−1^, 150 μL); (**f**) NIR-II FLI signal quantification in the tumor regions at different time points [45]. Copyright 2023, John Wiley and Sons.

In order to cope with the problem that fluorescent probes are mostly in “always-on” mode, Dai et al. [38] prepared a TME-activated multifunctional phototherapeutic nanoplatform based on a coordination-driven self-assembly strategy. The nanoplatform selected Fe(III) ions as cross-linking nodes, and coassembled carboxyl-modified Ag_2_S quantum dots (QDs) and ultra-small NIR-II semiconductor polymer dots (DBZ PDs) into a single nanoplatform (Ag_2_S-Fe(III)-DBZ nanoparticles, denoted as AFD NPs) (Figure 4a). AFD NPs have strong biosafety because of their TME-activating properties. AFD NPs are triggered to disassemble by GSH overexpressed in the TME by disrupting the metal ligand after entering the tumor cells, which facilitates deep tumor penetration and intracellularization, and reduces the side effects on normal tissues. In normal tissues, AFD NPs were maintained in the NIR-II fluorescence quenching state due to the Förster resonance energy transfer (FRET) effect between Ag_2_S and DBZ quantum dots. However, under 808 nm excitation, the AFD NPs responded to the overexpression of GSH in the TME and then were disassembled at the tumor site. The fluorescence responsiveness of Ag_2_S QDs was restored, which significantly improved the imaging sensitivity and signal-to-background ratio (SBR) of the AFD NPs. Fe(III) in AFD NPs significantly depletes GSH via redox reaction, and the resulting Fe(II) catalyzes endogenous H_2_O_2_ to generate cytotoxic ROS for CDT via the Fenton reaction. The depletion of GSH further enhances oxidative damage to the tumor and increases the efficiency of CDT. The intrinsic NIR-II PTT effect of DBZ PDs was enhanced by NIR-II light (1064 nm) excitation and thermally enhanced CDT. This nanoplatform is a diagnostic and therapeutic integrated composite phototherapeutic nanoplatform that can tumor-specifically activate NIR-II FLI and synergistically NIR-II PTT and CDT simultaneously, which provides an important experience for accurate diagnosis and combined therapeutic integration of tumors.

### 3.2. Metal Nanomaterials

Due to the advantages of controllability and high efficiency, the photocatalytic generation of H_2_O_2_ by metallic nanomaterials has been considered as a possible strategy to supply H_2_O_2_ in cells. It has been shown that magnetite nanoenzymes can activate the Fenton reaction to produce ROS, such as ·OH, which kills tumor cells through iron death (a programmed cell death dependent on iron ions) [95,96,97]. NIR-II-responsive nanoenzymes with photothermal conversion properties can take advantage of absorbing external light energy and converting it to heat to raise the local temperature to kill cancer cells [98]. Furthermore, the synergistic action of PTT and nanoenzymes-induced CDT not only combines the tumor-treatment capabilities of these two therapeutic modalities, but also provides additional therapeutic effects by further enhancing the activity of the Fenton reaction through the PTT-induced temperature increase [99,100,101]. Therefore, this PTT collaborative CDT tumor-treatment strategy based on metal nanomaterials has high research value. Li et al. [39]. discovered that NIR (808 nm) light can trigger intracellular H_2_O_2_ production by synthesizing MoSe_2_/CoSe_2_ nanosheets. NIR-II light (1000–1700 nm) has more potential in the biomedical field due to its excellent depth of penetration (~3 mm) and the maximum allowable exposure (1 W cm^−2^). Ma et al. [40] used a simple coprecipitation method to prepare Cu-Ni_0.85_Se@PEG nanocomposites for intracellular NIR-II photocatalytic production of H_2_O_2_. Density–functional theory calculations and experimental data confirmed that the metallic properties of Cu-Ni_0.85_Se make it a good candidate for photocatalysis. The metallic properties of Cu-Ni_0.85_Se give it good NIR absorption and photothermal conversion efficiency (59.6%, 1064 nm). The continuous inter- and intra-band jumps of the photothermal-assisted metal catalyst contribute to the enhancement of the redox capacity and the effective separation/transfer of charge, which is favorable for the generation of H_2_O_2_. Thus, the prominent metal properties endowed CuNi_0.85_Se@PEG with high photocatalytic activity. The nanomaterial also possesses peroxidase (POD), catalase (CAT) enzyme, and GSH oxidase activities, which can deplete intracellular GSH, disrupt the redox balance, and promote oxidative stress, making it a good multifunctional nanocomposite. 

Meng et al. [41] successfully synthesized a novel multifunctional nanoenzyme that provides synergistic tumor therapy by enhancing CDT through PTT. The nanoenzyme, called CEMNDs, has magnetic-carbon core–shell nanostructures and superparamagnetism, and exhibits excellent colloidal stability. The CEMNDs in TME activate the Fenton reaction to generate ·OH for CDT. Under near-infrared (1064 nm) irradiation, the CEMNDs exhibit an ideal photothermal conversion efficiency as high as 39.2%. Chen et al. [42] developed an endogenously activated iron(II)-activated nanomedicine, copper hexacyanoferrate (CuHCF), based on a Prussian blue copper analog [102], and for the first time served as a responsive agent for endogenous Fe^2+^ triggering of CDT and NIR-II PTT activation in situ. Endogenous Fe^2+^-triggered redox cycling of Cu and Fe active centers can promote interfacial electron transfer, which in turn facilitates ·OH generation via CuHCF-mediated Fenton/Fenton-like reactions. Interestingly, only when exposed to endogenous Fe^2+^, CuHCF showed strong NIR-II uptake, which could effectively mediate the synergistic treatment of tumor CDT and PTT and exhibit excellent antitumor capabilities. Therefore, metal nanomaterials synergizing tumor PTT and CDT via NIR-II light is a very promising strategy for tumor combination therapy.

### 3.3. Small Molecule Probe

Organic small-molecule NIR-II fluorophores have unique advantages, such as biocompatibility and safety, and have great potential for clinical applications [103]. However, due to the competition between radiative and nonradiative transitions, small molecule organic fluorophores often exhibit excellent fluorescence properties but lack efficient photothermal conversion efficiency [104,105]. Therefore, in order to realize the construction of more efficient diagnostic and therapeutic integrated nanoplatforms, the development of an organic NIR-II fluorophore with highly efficient and stable photothermal properties is still crucial research. Small-molecule organic fluorophores usually have two different aggregation states [106]. In general, the spatial stacking mode in which molecules are stacked in a staggered manner along the coplanar-parallel direction is called the J-aggregation state, and the spatial stacking mode in which molecules are stacked in a face-to-face overlapping manner along the coplanar-perpendicular direction is called the H-aggregation state [107]. Fluorophores in the J-aggregation state tend to acquire a redshift and improve the fluorescence spectrum in favor of FLI [108,109]. On the contrary, the H-aggregation state changes the electronic transition mode of the fluorophore from radiative to nonradiative transitions, which reduces the FLI effect of the fluorophore [110] but potentially improves the photothermal properties of the NIR-II fluorescent molecule. Yu et al. [43] constructed for the first time a loaded NIR-II-responsive fluorescent liposome (RRIALP-C4) loaded with IR-1061, and utilized its ingenious structure containing both aggregated and free states to achieve the integration of FLI and PTT effects. The nanomaterial utilizes anionic liposomes (IALPs) loaded with IR-1061 to encapsulate the chemotherapeutic drug Carboplatin, which is then modified with three different peptides, RGD (Arg-Gly-Asp), TAT48-60 (GRKKRRQRRPPQ), and RR9 (RGDRRRRRRRRC), to improve tumor targeting. RRIALP-C4 was successfully applied to PTT synergistic CDT under the guidance of in vitro and ex vivo NIR-II FLI and NIR-II photothermal imaging (PTI) (Figure 4b–d). This study innovatively developed a fluorescent liposome nanosystem with IR-1061 in both the aggregation state and the free state, which pointed out a new direction for the research of integrated nanoplatforms for tumor diagnosis and treatment.

Donor-acceptor-donor (D-A-D) organic small molecules are a class of photothermal transfer agents for combined CDT/PTT therapy with tunable optical–physical properties and good biocompatibility [111,112]. Under light irradiation, D-A-D small molecules can achieve both localized thermotherapy and NIR-II FLI for apoptosis and diagnosis of tumors. Wang et al. [44] prepared P(DPP-BT/DOX) nanoparticles based on D-A-D small molecule drug delivery for NIR-II FLI and PAI-guided PTT/CDT combination therapy. However, this delivery system is simply obtained by physically wrapping adriamycin (DOX) and hydrophobic D-A-D small molecules, which can greatly reduce the drug-carrying efficiency. Sun et al. [45] successfully constructed water-soluble BTP/DOX/2DG nanoparticles for NIR-II FLI and PAI for combination therapy of CDT, PTT, and starvation therapy in tumors. Phenylboronic acid (PBA)-modified water-soluble D-A-D molecules (BBT-TF-PBA) were first synthesized as drug carriers for encapsulating DOX and the glycolysis inhibitor 2-deoxy-d-glucose (2-DG), and then physically encapsulated using DSPE-PEG2000. Upon reaching the low pH hypoxic TME, donor–acceptor ligand interactions and PBA-diol bonds were disassembled and simultaneously released DOX and 2-DG from BTP/DOX/2DG nanoparticles, which triggered CDT and starvation therapy. Under light excitation at 1064 nm, the BTP/DOX/2DG nanoparticles could convert light energy into thermal energy, realizing effective imaging diagnosis of NIR-II PTT, FLI, and PAI (Figure 4e,f).

## 4. Photothermal Therapy Synergized with Thermodynamic Therapy

With the help of nanomaterials, the PTT of NIR-II can be combined with multiple therapeutic approaches to improve the therapeutic effect [113]. Although PTT can work synergistically with PDT, TME is hypoxic, which limits the therapeutic efficacy of treatments that depend on oxygen levels. Fortunately, thermodynamic therapy (TDT) has recently been utilized as an alternative treatment modality, which utilizes oxygen-independent free radicals (e.g., carbon-radicals) to kill tumors and displace ROS [114]. Carbon-radicals, which are produced by azo compounds triggered by heat or irradiation stimulation, are not only used for radical polymerization, but are also used in the application of inducing oxidative stress in biological systems [115], which can be a good substitute for PDT to work synergistically with PTT.

### Polymers

Organic conjugated polymers are an ideal NIR-II-absorbing material, where the radiative excursion of fluorophores under light stimulation can produce fluorescence emission for tumor imaging [116,117], and the nonradiative excursion can produce photothermal effect for tumor therapy [118,119]. However, the application of polymers has been facing some biosafety limitations, such as poor water solubility and aggregation in aqueous media, leading to aggregation-caused quenching (ACQ) [115]. Therefore, the AIE technology has emerged with a promising development, providing a new design perspective and direction for the development of NIR-II FLI technology [120]. Unlike ACQ fluorophores, AIE luminophores emit weakly or not in the solution state, but fluorescence is significantly enhanced in the aggregated state [121]. Aggregation-induced emission luminous agents (AIEgens) can not only be used in FLI, but also have potential applications in PTT [122]. Therefore, the development of fluorophores with AIE properties of NIR-II absorption will provide new ideas for integrated nanoplatforms for tumor diagnosis and treatment.

**Figure 5:**
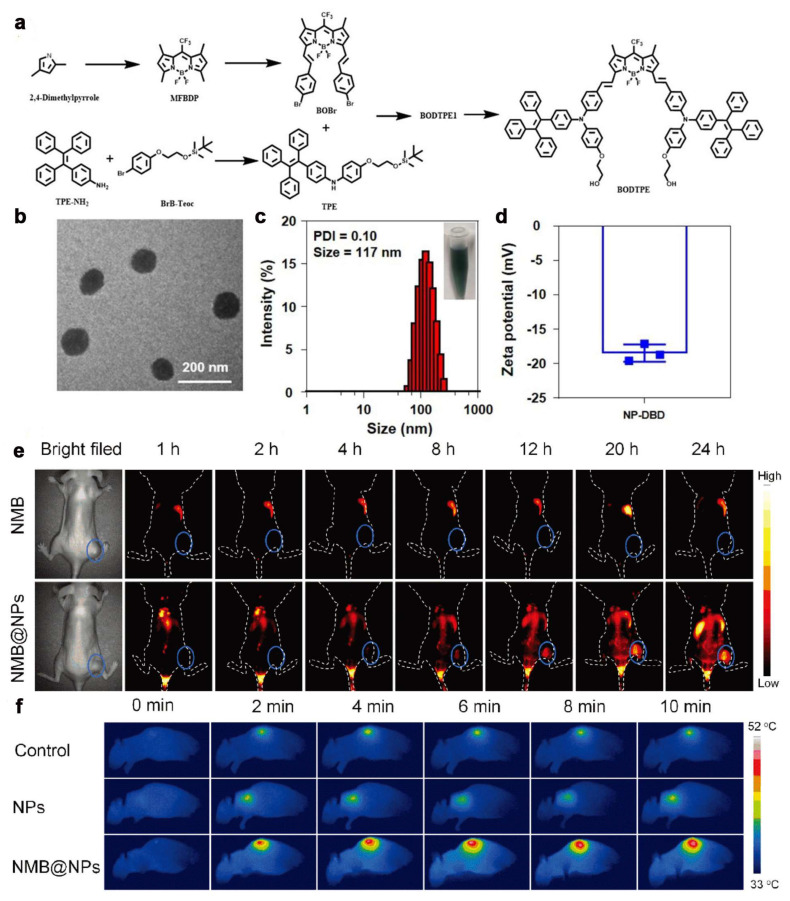
(**a**) Synthetic routes of BODTPE; (**b**) Characterization of NP-DBD by transmission electron microscopy (TEM); (**c**) The hydrodynamic size distribution of NP-DBD; (**d**) Mean zeta potential of NP-DBD [48]. Copyright 2023, Adv. Mater. (**e**) In vivo NIR-II FL imaging results of free NMB and NMB@NPs at different time intervals; (**f**) Real-time infrared thermal images of oral cancer xenograft model mice [46]. Copyright 2023, John Wiley and Sons.

Zhang et al. [46] constructed a NIR-II FLI-guided PTT synergistic TDT tumor therapy system, in which azo compound-containing NPs were prepared by one-pot copolymerization of carbon radical monomers, ethyl 2,6-diisocyanatohexanoate, and PEG molecules, and AIEgen was loaded as a molecular heater in the hydrophobic core (NMB@NPs) (Figure 5e,f). Under 808 nm laser irradiation, NMB@NPs controlled the balance between fluorescence and photothermal effects. AIEgens-based NIR-II FLI can effectively localize the tumor site and monitor the treatment process in real time, showing high detection sensitivity, resolution, and imaging depth. In a patient-derived xenograft (PDX) mouse model of oral cancer, the photothermal effect of NMB@NPs and the thermally triggered release of carbon radicals from azo polymers showed high tumor inhibition without severe systemic toxicity. The successful application of AIEgens allows nanomaterials to simultaneously have the ability of diagnostic and therapeutic integration, which is likely to open up a new field in precision medicine. Notably, Zhuang et al. [47] developed a NIR-II-responsive anti-hypoxic PS (designated as TPEQM-DMA) by combining specific spectral properties and unique bioactivities into a single organic molecule. The nanomaterial is derived from a tetraphenylethylene (TPE) backbone with AIE activity, which allows for the formation of brighter fluorescence in the aggregated state. The enhancement of the π-conjugated push–pull effect promotes the emission of TPEQM-DMA in the NIR-II region (>1000 nm). Under white light irradiation, TPEQM-DMA produced O_2_^·−^ and ·OH only by type I photochemical processes. The combination of NIR-II fluorescence and type I photosensitization enabled efficient NIR-II FLI-guided PDT of TPEQM-DMA NPs in vivo. Cui et al. [48] described the development of a drug delivery system, NP-DBD. NP-DBD is mainly composed of two core components: the photosensitizer BODTPE and the targeting ligand DBCO (Figure 5a–d). BODTPE, as a novel small-molecule PS, showed excellent performance in the inclusion of the AIE effect in the PDT, PTT, and NIR-II fluorescence showed excellent performance. This proves that AIEgens can not only synergize with PTT, but also combine with other tumor therapeutics, which has a broad outlook for application.

## 5. Photothermal Therapy Synergistic Immunotherapy

In recent years, cancer IMT has been positioned as a tumor-treatment modality alongside surgery, chemotherapy and radiotherapy, not only to treat local and distant metastatic tumors, but also to inhibit tumor recurrence [123]. Tumor vaccines, ICD, and pattern recognition receptor (PRR) stimulators have become effective means of activating the tumor immune system [124]. However, the response rate of IMT is quite low, ranging from 10% to 30% for different types of tumors [125]. The delivery of immune checkpoint inhibitors, cytokines, and vaccine adjuvants to the targeting site using a nanomedicine delivery system has been used to modulate immunosuppressive TME [126]. However, conventional nanomedicine delivery systems are not controlled and may activate self-antigen-reactive T cells after delivery, which may cause side effects [127]. In contrast, light-activated nanomedicines have been used to deliver immunotherapeutic agents that can more selectively and specifically reprogram immunosuppressive TME [128,129,130]. PTT also induces ICD in cancer cells, typically releasing CRT, HMGB1, and ATP into the extracellular compartment, thereby recruiting immune cells [131]. Moreover, NIR-II-responsive nanomaterials have greater tissue penetration depth and lower phototoxicity, which can combine IMT with PTT for efficient tumor treatment.

### 5.1. Polymers

According to recent studies, the reason why the immune response rate of IMT is relatively low may be related to the immunosuppressive microenvironment of tumor tissues, such as low tumor immunogenicity, rejection of T cells, and high expression of immunosuppressive molecules such as programmed death ligand (PD-L1), etc. [132,133]. In addition, the extracellular matrix (ECM) of solid tumors is very dense, which can act as a natural physical barrier, hindering the penetration and diffusion of anticancer drugs. Immune cells are also unable to function properly, resulting in a low IMT immune response rate [134]. Therefore, the construction of nanoplatforms that can regulate the immunosuppressive microenvironment of tumor tissues is the key to improving the anti-cancer efficiency of IMT. Li et al. [49] reported a NIR-II photoactivated semiconductor polymer nanoparticles (SPNs) loaded with NO precursor (L-arginine) and PD-L1 inhibitor and coated with a bromelain-modified thermo-responsive shell. The nanomaterial has a decent biosafety profile, and upon entry into tumor cells, localized heat is generated under 1064 nm laser irradiation, leading to the disruption of the thermo-responsive shell to control the release of L-arginine and PD-L1 inhibitors. The nanomaterial has the ability to modulate the immunosuppressive tumor microenvironment through a trinity of strategies, including degradation of the ECM to promote NPs accumulation and immune cell infiltration, triggering of the ICD through NIR-II PTT and GT to enhance tumor immunogenicity, and blocking of PD-L1 immunosuppression through a PD-L1 inhibitor. This trinity of NIR-II photoactivated organic nanoplatform-mediated combination therapy not only has higher efficacy, but also can be loaded with other gas deliverers such as hydrogen sulfide (H_2_S) and carbon monoxide (CO) because of its flexibility.

### 5.2. Small Molecule Probe

**Figure 6:**
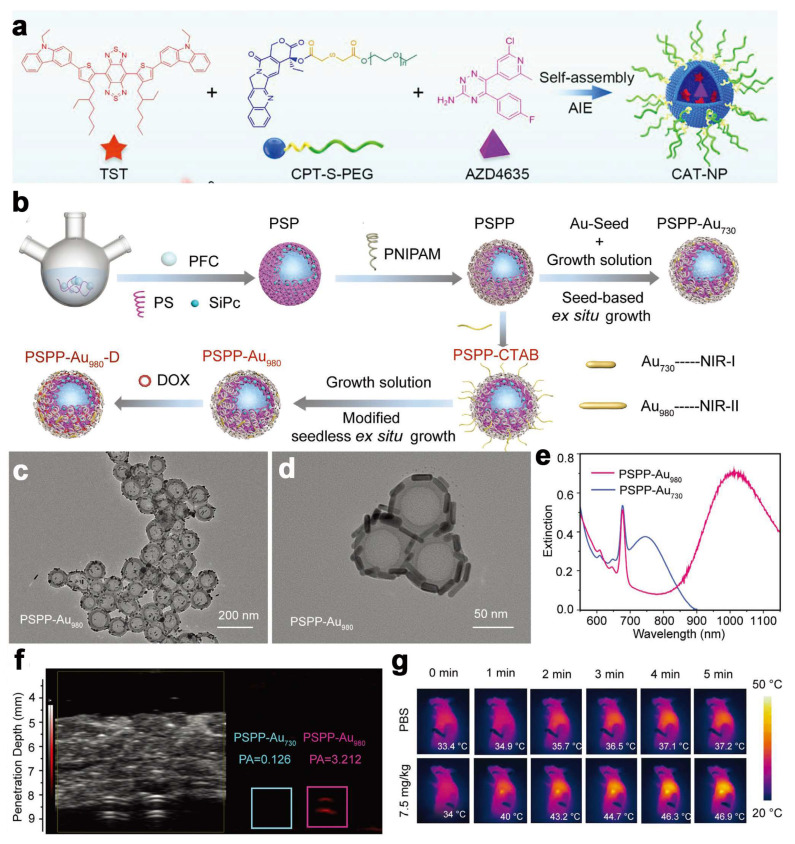
(**a**) The TST, CPT-S-PEG, and AZD4635 were co-assembled into nanoparticles (CAT-NP) through nanoprecipitation method [50]. Copyright 2022, John Wiley and Sons; (**b**) Schematic illustration of the synthesis of PSPP-Au_980_-D or PSPP-Au_730_; (**c**) TEM image and (**d**) Corresponding magnified TEM image of PSPP-Au_980_; (**e**) Extinction spectra of PSPP-Au_980_ and PSPP-Au_730_, measured with UV–vis spectroscopy; (**f**) In vitro PA imaging of PSPP-Au_980_ and PSPP-Au_730_ at 4 mm tissue depth. The corresponding signal was obtained at 960 or 730 nm by PA imaging; (**g**) IR thermal images of MIA PaCa-2 tumor-bearing mice after the IP injection of PBS or PSPP-Au_980_-D (7.5 mg kg^−1^) under 980 nm laser irradiation (0.1 W cm^−1^) [55]. Copyright 2022, John Wiley and Sons.

In order to solve the current problem of commercial fluorescent dyes that are prone to ACQ and shallow fluorescence penetration during in vivo delivery, Wang et al. [50] synthesized a novel NIR-II-responsive nanomaterial with AIE functionality and photo-thermal functionality, named TST. Comedophylline (CPT), a cytotoxic quinoline alkaloid with a large number of conjugated double bonds, was used as an amphiphilic compound with redox-sensitive monosulfide bonds in the middle. The authors first linked CPT and a polyethylene glycol ester by esterification reaction to form an amphiphilic compound with a redox-sensitive monosulfide bond in the middle (CPT-S-PEG), and then prepared nanoparticles by coassembling CPT-S-PEG, TST, and a novel immune checkpoint inhibitor, AZD4635, using the nanoprecipitation method [135]. (Figure 6a) Due to the strong interaction between CPT and TST, the nonradiative attenuation of TST was inhibited, resulting in strong NIR-II fluorescence when the nanoparticles were intact, as a means to perform NIR-II imaging. PTT induces ICD in tumor cells, releasing large amounts of ATP into the TME, which in turn recruits immune cells for IMT. However, the excess ATP is converted to adenosine via the CD39-CD73-A2AR pathway, which inhibits immune cell activity. At this point, the nanoparticles released by the photothermal disintegration of AZD4635 precisely blocked this pathway, achieving a good synergistic effect of PTT, CDT, and IMT. 3,3′,5,5′-Tetramethylbenzidine (TMB) is a safe chromogenic reagent, which has been widely used in clinical studies for free radical detection and blood biochemical tests [136]. Its oxidation product exhibits broadband absorption at NIR-II (750–1200 nm) and responds specifically to pH changes and H_2_O_2_, an essential redox substance in TME. Pu et al. [51] packaged a prephotothermic agent of TMB, a generator of H_2_O_2_, and a starvation therapeutic mediator of GOD in a nanoliposome (denoted as LGT). GOD effectively catalyzes the conversion of endogenous glucose into gluconic acid and H_2_O_2_, which further reacts with the prephotothermolysis agent TMB to form an “on” NIR-II-absorbing and biodegradable charge transfer complex (CTC) for synergistic PTT in tumor-starvation therapy. This smart nanoplatform triggers a vaccine-like immune response after in situ photothermal ablation of primary tumors and further binds to anti-CTLA-4 checkpoint blockade, which activates photothermal immunotherapy to inhibit distant tumor growth and cancer metastasis via starvation therapy. This diagnostic and therapeutic integrated nanoplatform can not only guide clinical tumor therapy through the NIR-II PAI imaging capability yet hopefully, but also synergize the starvation therapy/PTT/IMT, demonstrating an extreme antitumor capability. Xu et al. [52] introduced upconversion nanoparticles (UCNPs) and IR-1048 dyes into lipo aptamer nanostructures to construct a novel NIR-II (1000–1700 nm) therapeutic nanoplate, UCILA, which possesses both enhanced five-mode NIR-II imaging capability and good 1064 nm near-infrared (NIR) photothermal therapeutic capability for precise detection and treatment of lung cancer. The spherical UCILA enhanced photoacoustic imaging up to 4.5 cm depth with a high signal-to-noise ratio in the NIR-II window. The photothermal conversion rate of UCILA after coincubation with A549 cells was 31.3%. Thus, it can be seen that small molecule fluorescent probes are creating a buzz in NIR-II imaging-guided tumor synergistic therapy.

## 6. Photodynamic Therapy Synergized Chemodynamic Therapy

CDT has been widely studied for a long time, mainly through the Fenton/Fenton-like reaction, using chemotherapeutic drugs to catalyze the production of ∙OH from H_2_O_2_ in tumor cells, which disrupts the redox balance and leads to cell death. However, the level of H_2_O_2_ inside the tumor is limited, so the effect of CDT alone is far from expected. To improve its tumor therapeutic efficiency, an additional supply of H_2_O_2_ is needed to promote CDT. According to the previous section, PDT is a clinically approved noninvasive antitumor strategy that destroys malignant cancer cells by activating PSs through UV or visible light to generate ROS. Both CDT and PDT can utilize H_2_O_2_ in TME to kill tumor cells. If a nanoplatform can be constructed to increase the H_2_O_2_ content in TME, the tumor therapeutic ability of CDT and PDT can be greatly exploited.

### 6.1. Rare Earth-Doped Nanoparticles

In recent years, nanocomposites based on metal-organic frameworks (MOFs) have offered many possibilities for designing multifunctional nanoplatforms in the field of cancer therapy [137]. ZIF-8 is a typical MOF that has been widely used in the fields of bioimaging and cancer therapy due to its extremely low systemic toxicity and sensitive pH-responsive behavior. ZIF-8, as a photoresponsive nanomaterial, can be excited by UV to produce ·O^2−^, and thus realize photocatalytically driven PDT. However, ZIF-8 has a relatively large band gap and responsiveness to UV, and cannot be directly excited by NIR-II, which limits its application in the field of photocatalytic anticancer. In this regard, researchers have reported many rare earth doping methods to improve this situation.

Rare earth-doped upconversion (UC) nanoparticles have attracted considerable interest in anticancer applications due to the realization of NIR-to-UV-visible light conversion [138]. Rare earth-doped UC NPs not only play a key role through photon-triggering mechanisms but also serve as potential optical probes for UC FLI [56]. However, the application of UC fluorescence in optical imaging is limited by the limited tissue penetration depth, low contrast, and poor spatial resolution. In recent years, lanthanide-doped down-conversion (DC) nanomaterials (LDNPs) with NIR excitation have become increasingly popular for high-sensitivity in vivo optical imaging due to their emission in NIR-II. Li et al. [53] designed and prepared NIR photoresponsive LDNPs@Fe/Mn-ZIF-8 nanomaterials via a self-assembly strategy of Fe^2+^/Mn^2+^/Zn^2+^ with 2-methylimidazole on the surface of LDNPs-polyvinylpyrrolidone (PVP), realizing the synergistic catalytic therapy of PDT/CDT under NIR imaging guidance. Notably, the double doping of Fe^2+^/Mn^2+^ not only extends the absorption range of the ZIF-8 photosensitizer in the UC emission spectrum of LDNPs, but also provides a Fenton/Fenton-like reagent for CDT through the tumor-responsive degradation of Fe/Mn-ZIF-8. Fe^3+^/Mn^3+^/Mn^4+^ scavenges the overexpression of TME with GSH and H^+^, thus protecting the generated ROS (including ·O^2−^ and ·OH) from being scavenged by GSH and enhancing the anticancer effect of CDT/PDT. Degradation of Fe/Mn-ZIF-8 gives the nanomaterial a tumor self-enhancing NIR-II FLI capability, which can provide accurate guidance and monitoring of the tumor-treatment process for PDT/CDT.

### 6.2. Small Molecule Probe

NIR-II-responsive small molecule fluorescent probes are being widely used in the construction of integrated nanoplatforms for tumor diagnosis and treatment to achieve synergistic tumor PTT, PDT, and CDT therapy. PROteolysis TArgeting Chimeras (PROTACs) is an emerging medical approach that induces proteolytic hydrolysis of target proteins by recruiting proteins of interest (POIs) to the E3 ubiquitin ligase, and subsequently labeling the proteins by the addition of ubiquitin for protease-mediated destruction. Hu et al. [54] successfully prepared PROTAC-Cy7@BSA nanoparticles, used for in vivo NIR-II imaging, noninvasive observation of tumor vascular networks, and intraoperative real-time navigation of tumor dissection. PROTAC-Cy7 was constructed through a trinity molecular design strategy. It integrates the MCL-1-bound warhead, the E3 ligase ligand pomalidomide, and the heptamethine cyanine dye connecting the warhead and the ligand into the same scaffold, and wraps PROTAC-Cy7 with bovine serum albumin (BSA). With this assembly strategy, PROTAC-Cy7 not only improves its optical stability and solubility in water, but also greatly enhances the specific accumulation at the tumor site, contributing to the clear identification of blood vessels, tumors, and adjacent boundaries. 

### 6.3. Precious Metals

Hypoxia is an inherent feature of many malignant solid tumors. Perfluorocarbon (PFC) fluids have very low intermolecular interactions and high oxygen solubilization capacity [139]. Various PFC-based nanostructures have been investigated for the elimination of hypoxic tumors. For example, lipid-encapsulated PFCs and PSs have been used to improve the efficacy of PDT [140]. However, PFCs still cannot avoid the problem of premature leakage before delivering oxygen to the hypoxic region. Triggering oxygen release with NIR-II light with lower energy loss is a good method. Gold nanorods (AuNRs) as a noble metal PTA have been reported to optically trigger liquid PFC nanodroplets in ultrasound and photoacoustic imaging. In addition, the extinction peaks of AuNRs can be tuned to the NIR-II region by several methods, such as post-tuning, surface functionalization, and noble metal binding [141]. Therefore, AuNRs are considered to be the preferred photothermal excitants for PFC-based oxygen control systems. Zhang et al. [55] designed a photothermally controlled “oxygen bomb” PFC/SIPC@PS@PNIPAM-AU_980_-DOX (PSPP-AU_980_-D) (Figure 6b–e). The PSPP-AU_980_-D is realized by encapsulating the PFC core in a functionalized double-layer polymer housing. The authors prepared AuNRs with an extinction peak of 980 nm in the polymer PNIPAM outer layer by doping silicon phthalocyanine (SIPC) of PSs with an extinction peak located at 680 nm into the polystyrene (PS) interlayer for PDT and combining the chemotherapeutic drug DOX in the polymer polyisopropylacrylamide (PNIPAM) outer layer by a modified two-step nucleated in situ growth process. The photothermal conversion efficiency of PSPP-AU_980_-D at 980 nm was 40.62%, with an oxygen-carrying capacity of 2.25 ± 0.22 mg g^−1^. Due to the good tissue penetration capability within the NIR-II biological window (980 nm), the temperature rise of the PSPP-AU_980_ solution under irradiation of 980 nm was up to 14.1 °C with a penetration depth of 4 mm, which is much higher than that of 9 °C under 730 nm excitation under the same conditions (Figure 6f,g). This novel design concept of PSPP-AU_980_-D is expected to provide a new pathway and opportunity for hypoxic tumor therapy in the future clinic.

## 7. Other

From the main research results in the past three years, PTT, PDT, CDT, and IMT are still the mainstream of antitumor research. Through the selection of suitable nanomaterials, the construction of a nanoplatform for tumor NIR-II imaging and combination therapy is still a promising therapeutic strategy for clinical application. However, there is no lack of scholars who dare to open up new ideas, combining enzyme therapy, starvation therapy, gene therapy, acoustic kinetic therapy, thermodynamic therapy, gas therapy, and other tumor therapies, which provides valuable experience in the development of integrated nanoplatforms for tumor diagnosis and treatment. Although few results have been reported now, it is still of great research value.

### 7.1. Rare Earth-Doped Nanomaterials

As mentioned above, LDNPs with NIR excitation have been widely studied due to their application in NIR-II in recent years. Wang et al. [56] constructed a novel erbium-doped (Er^3+^)-based NaLnF_4_@MOF core@shell heterostructure, which integrates near-infrared photodynamics and NIR-II imaging into tumor therapy. The nanomaterial can generate both upconversion luminescence (UCL) and NIR luminescence with a typical upconversion part and an optimized downconversion part. Under 980 nm laser irradiation, MOF obtains UCL from NaLnF_4_ via resonance energy transfer, triggering ^1^O_2_ production to destroy tumor cells. NIR-II imaging was also performed using downshifted emission at ~1530 nm to monitor the location and distribution of NPs, to allow accurate PDT treatment at lower power (0.12 W/cm^2^). Additionally, the synergistic effect of PDT and IMT, complemented by anti-programmed death ligand 1 (α-PD-L1), not only eradicated the primary tumor, but also inhibited the distant tumors with an effective tumor suppression rate of 95%.

### 7.2. Small Molecule Probe

To improve the efficiency of cancer treatment, mitochondria-targeted PDT, IMT, and related combination therapies have attracted widespread attention. However, most of the current mitochondria-targeted therapeutic organic small molecule probes emit light in the visible (400–750 nm) or NIR-I (750–1000 nm) region [142]. The development of mitochondria-targeted multifunctional nanoplatforms for NIR-II imaging guidance is a new focus. Yang et al. [143] identified an excellent organic dye, IR-FE, with a quantum yield (QY) of up to 31% in toluene. Yang et al. [57] further identified triphenylphosphine PEGylation (PEG2000-TPP) modification, and successfully synthesized a mitochondria-targeted organic photosensitizer, FEPT, for NIR-II-guided mitochondria-targeted synergistic PTT/PDT/IMT. Under the excitation of 808 nm laser light, FEPT could obtain a significant NIR-II fluorescence signal (QY: 1.64% in water), which helps to perform NIR-II imaging in vivo, locate the tumor position in real-time, and monitor the treatment process. In addition, FEPT has a high photothermal conversion efficiency (56.8%) and generates a large amount of ROS under 808 nm laser irradiation, which can easily lead to mitochondrial dysfunction in vivo, induce a powerful ICD, and activate specific tumor-immune response in vivo, giving full play to the synergistic effect of PTT/PDT/IMT, and ultimately leading to the death of cancer cells.

## 8. Conclusions and Outlook

Nanomaterials not only have optical properties, such as high MEC, low quantum yield, good photostability, and NIR absorption, but also have long blood circulation time. The use of nanomaterials for tumor imaging and therapy has been a popular choice for current research. Compared with NIR-I light, NIR-II light has significant advantages, such as low energy consumption, more desirable tissue penetration depth (up to 3.5 mm), better spatial resolution, higher sensitivity, etc., and has a broader application prospect in tumor imaging and therapy. Therefore, the development of NIR-II-responsive nanomaterials is a “win–win” strategy. With the progress of research in recent years, more and more NIR-II-responsive nanomaterials have been discovered, but in the past three years, the most frequently used are precious metals, polymers, organic small molecule dyes, rare earth-doped nanoparticles, metal nanomaterials, etc. However, these materials do not work alone, because a large number of studies have shown that the results that can be achieved by single tumor therapies are often unsatisfactory, and combined tumor-treatment strategies have a brighter future. Combining NIR-II-responsive nanomaterials with combined tumor therapy strategies can not only lead to higher tumor therapy efficiency, but also take advantage of the nanomaterials’ self-contained FLI and PAI capabilities, thus integrating tumor localization, diagnosis, and treatment. The development of such NIR-II-responsive integrated nanoplatforms for tumor diagnosis and treatment has been a popular research direction in the field of tumor therapy in the last three years. This paper summarizes the types of NIR-II-responsive tumor diagnostic and therapeutic integration nanoplatforms mainly developed by scholars in the last three years for combined tumor-treatment strategies, and succinctly introduces the mechanisms of various materials in FLI and PAI imaging and combined treatment.

Despite the exciting results of the NIR-II-responsive integrated nanoplatform for tumor diagnosis and treatment, there are still several issues that need to be systematically investigated and resolved before it can be pushed into clinical applications. (1) Although many studies have argued about the toxicity of nanomaterials, proving that they can be rapidly metabolized in vivo, and even claiming that their materials are completely nontoxic and harmless to the human body. However, the depth of biosafety studies in these research studies is still insufficient and the time span is too short to demonstrate whether they have long-term chronic toxic effects. In addition, it is too early to conclude by relying only on mouse transplantation tumor models as a method of evaluation, because mouse models are hardly representative of the real situation in the patient’s body. Therefore, we should continue to choose some animal models that are close to human relatives to evaluate the biosafety of nanomaterials in a long-term and chronic manner. Only in this way can its clinical application be promoted. (2) Although NIR-II light is better than NIR-I light, its penetration depth is still insufficient. Moreover, the reported laser excitation sources are still limited to below 1100 nm, which seriously affects the quality of tumor imaging and clinical applications. (3) The construction of some nanomaterials is still cumbersome, and the process needs to be optimized step by step if we want to push it into clinical applications.

In summary, the research of NIR-II-responsive tumor diagnosis and treatment integrated nanoplatform still has a long way to go before clinical application. However, at this stage, the diagnostic–therapeutic integrated nanoplatform has already demonstrated excellent tumor imaging and therapeutic effects and still has a very bright application prospect. As long as future work can be committed to breaking through this part of the limitations, we can promote the nanoplatform further toward clinical application.

## Figures and Tables

**Table 1:** Summary of Nanomaterials for tumor NIR-II imaging and synergistic therapy.

Types	NPs	Laser Source	Particularities	Therapeutic Applications	Refs.
polymers	FE-T	808 nm	4T1; NIR-II FLI	PTT/PDT/IMT	[32]
Small molecule probes	IR808	808 nm	colorectal cancer; NIR-II FLI	PTT/PDT	[33]
Small molecule probes	Y16-Pr-PEG	808 nm	4T1; NIR-II FLI and PAI	PTT/PDT	[34]
Small molecule probes	BTP-4F-DMO	808 nm	4T1; NIR-II FLI and PAI	PTT/PDT	[35]
Metal nanomaterials	C-DTTP	808 nm	MDA-MB; NIR-II FLI and PAI	PTT/PDT	[36]
Metal nanomaterials	Ag_3_SbS_3_	1064 nm	4T1; NIR-II PAI and MRI	CDT/PTT/PDT	[37]
Polymers	Ag_2_S	808 nm	4T1;NIR-II FLI	PTT/CDT	[38]
Metal nanomaterials	MoSe_2_/CoSe_2_@PEG	808 nm	NIR-II MRI and PTI	PTT/CDT	[39]
Metal nanomaterials	Cu-Ni_0.85_Se@PEG	1064 nm	NIR-II MRI	PTT/CDT	[40]
Metal nanomaterials	CEMNDs	1064 nm	4T1	PTT/CDT	[41]
Metal nanomaterials	CuHCF	1064 nm	4T1	PTT/CDT	[42]
Small molecule probe	IR-1061	1061 nm and 808 nm	NIR-II PTI and FLI	PTT/CDT	[43]
Small molecule probe	DPP-BT	730 nm	Hela; NIR-II PAI and FLI	PTT/PDT/CDT	[44]
Small molecule probe	BTP/DOX/2DG	1064 nm	143B; NIR-II PAI and FLI	PTT/CDT	[45]
Polymers	NMB@NPs	808 nm	PDX; NIR-II FLI	PTT/TDT	[46]
Polymers	TPEQM-DMA	>1000 nm	MCF-7; NIR-II FLI	PDT/ Ferroptosis	[47]
Polymers	BODTPE	808 nm	4T1; NIR-II FLI	PDT/PTT/IME	[48]
Polymers	SPNs	1064 nm	4T1; NIR-II FLI	PTT/IME/GT	[49]
Small molecule probe	TST	808 nm	4T1; NIR-II FLI and PAI	PTT/IME/CDT	[50]
Small molecule probe	LGT	1064 nm	4T1; NIR-II FLI and PAI	PTT/IME	[51]
Small molecule probe	UCILA	1064 nm	A549; five-mode NIR-II imaging	PTT/IME	[52]
Rare earth-doped nanoparticles	LDNPs@Fe/Mn-ZIF-8	980 nm	NIR-II FLI	PDT/CDT	[53]
Small molecule probe	PROTAC-Cy7	808 nm	CT26; NIR-II FLI	PTT/PDT/CDT	[54]
Precious metals	PSPP-AU_980_-D	680 and 980 nm	Orthotopic pancreatic tumor; NIR-II FLI and PAI	PTT/PDT/CDT	[55]
Rare earth-doped nanoparticles	NaLnF4@MOF	980 and 1530 nm	CT26; NIR-II imaging	PDT/IMT	[56]
Small molecule probe	FEPT	808 nm	4T1; NIR-II FLI	PTT/PDT/IMT	[57]

## Data Availability

Not applicable.

## Abbreviations

·OH	hydroxyl radicals
^1^O_2_	singlet oxygen
2-DG	2-deoxy-d-glucose
α-PD-L1	anti-programmed death ligand 1
ACQ	aggregation-caused quenching
A-D-A	acceptor-donor-acceptor
A-DA’D-A	“receiver-donor -Acceptor’-Donor-Acceptor”
AFD NPs	Ag_2_S-Fe(III)-DBZ nanoparticles
AIE	aggregation-induced emission
AIEgens	aggregation-induced emission luminous agents
ATP	adenosine triphosphate
AuNRs	Gold nanorods
BSA	bovine serum albumin
CAT	catalase enzyme
CDT	chemodynamic therapy
CPT	comedophylline
CRT	calreticulin
CT	computed tomography
CTC	charge transfer complex
CuHCF	copper hexacyanoferrate
D-A-D	donor-acceptor-donor
DBZ PDs	ultra-small NIR-II semiconductor polymer dots
DC	down-conversion
DOX	adriamycin
EPR	enhanced permeability and retention
FLI	fluorescence imaging
FRET	Förster resonance energy transfer
GSH	glutathione
GT	gas therapy
H_2_O_2_	hydrogen peroxide
HMGB1	high-mobility-group protein 1
IALPs	anionic liposomes
ICD	immunogenic cell death
IMT	immunotherapy
LDNPs	lanthanide-doped down-conversion nanomaterials
LGT	nanoliposome
MEC	molar extinction coefficient
MOFs	metal-organic frameworks
MPE	maximum permissible skin exposure
MRI	magnetic resonance imaging
NIR-I	first near-infrared window (wavelengths of 750–1000 nm)
NIR-II	near-infrared second region
NPs	nanoparticles
PA	photoacoustic
PAI	photoacoustic imaging
PBA	phenylboronic acid
PD-L1	programmed death ligand
PDT	photodynamic therapy
PDX	patient-derived xenograft
PEG	polyethylene glycol
PEG2000-TPP	PEGylation
PFC	perfluorocarbon
PNIPAM	polyisopropylacrylamide
POD	peroxidase enzyme
POIs	proteins of interest
PROTACs	PROteolysis TArgeting Chimeras
PRR	pattern recognition receptor
PS	polystyrene
PSs	photosensitizers
PTAs	photothermal agents
PTI	photothermal imaging
PTT	photothermal therapy
PVP	polyvinylpyrrolidone
QDs	quantum dots
QY	quantum yield
ROS	reactive oxygen species
RRIALP-C4	fluorescent liposome
SBR	signal-to-background ratio
SCCs	supramolecular coordination complexes
SIPC	silicon phthalocyanine
SPNs	semiconductor polymer nanoparticles
TDT	thermodynamic therapy
TMB	3,3′,5,5′-Tetramethylbenzidine
TME	tumor microenvironment
TPE	tetraphenylethylene
UC	upconversion
UCL	upconversion luminescence
UCNPs	upconversion nanoparticles
